# Biventricular thrombi in dilated cardiomyopathy in a patient with human immunodeficiency virus infection: a case report

**DOI:** 10.1186/s13104-015-1140-x

**Published:** 2015-04-28

**Authors:** Clovis Nkoke, Liliane Mfeukeu Kuate, Engelbert Bain Luchuo, Sandrine Dikosso Edie, Jerome Boombhi, Alain Menanga

**Affiliations:** Faculty of Medicine and Biomedical Sciences, University of Yaounde I, Yaounde, Cameroon; Cardiology Unit, Yaounde Central Hospital, Yaounde, Cameroon; Centre for Population Studies and Health Promotion, CPSHP, Yaounde, Cameroon; Department of Military Health, Ministry of Defense, Yaounde, Cameroon

**Keywords:** Dilated cardiomyopathy, Human immunodeficiency virus, Biventricular thrombi, Sub-Saharan Africa

## Abstract

**Background:**

Sub-Saharan Africa is undergoing epidemiological transition with an increase in the prevalence of cardiovascular diseases that will add to the already devastating burden of infectious diseases such as human immunodeficiency virus infection. Human immunodeficiency virus infection is increasingly being recognized as an important etiological factor for dilated cardiomyopathy with the potential complication of intraventricular thrombus. However, biventricular thrombi are extremely rare. We report on a rare finding of biventricular thrombi in dilated cardiomyopathy in a patient with human immunodeficiency virus infection in Cameroon.

**Case presentation:**

A 52-year old Cameroonian male patient with human immunodeficiency virus infection since 4 years, longstanding heavy alcohol consumption and cigarette smoking presented with gradually worsening shortness of breath, fatigue, persistent dry cough and lower extremity swelling of about two weeks duration. Congestive heart failure was diagnosed. Echocardiography showed left ventricular chamber enlargement with severe left ventricular systolic dysfunction and biventricular thrombi. The thrombi were immobile and regular in configuration, suggesting they were old. He was treated with a conventional heart failure treatment including loop diuretics and angiotensin converting enzyme inhibitors and anticoagulants for the biventricular thrombi. Six months later, a control echocardiography showed a significant decrease in the size of the thrombi. There was no evidence of systemic or pulmonary embolization during follow up.

**Conclusion:**

Dilated cardiomyopathy may be seen in patients with human immunodeficiency virus infection, although other mechanisms needs to be assessed, but the occurrence of biventricular thrombi is rare.

## Background

Sub-Saharan Africa (SSA) faces a double burden of communicable diseases such as human immunodeficiency virus (HIV) infection and non communicable diseases. Among the several causes of dilated cardiomyopathies, HIV infection is increasingly being recognized as an important cause of acquired dilated cardiomyopathy [1,2]. In a recent report from India, 17.6% of HIV infected patients were found to have dilated cardiomyopathy [3]. Dilated cardiomyopathy in HIV infected patients is associated with a poor prognosis with a median survival of 101 days, as compared with 472 days in patients with a normal heart who are at a similar stage of disease [4]. Left ventricular thrombus is a well described and common complication in dilated cardiomyopathies. However, the occurrence of biventricular thrombi is extremely rare and only few cases have been reported in SSA [5]. This simultaneous presence of right ventricular and left ventricular thrombi increases the risk of both pulmonary and systemic embolization. In the following case, a patient with history of HIV infection, heavy alcohol consumption and cigarette smoking presented with congestive dilated cardiomyopathy complicated with biventricular thrombi.

## Case presentation

A 52-year old Cameroonian male patient with history of HIV infection diagnosed four years earlier, current cigarette smoking and heavy alcohol consumption for more than ten years was referred to the cardiology department of the Yaounde Central hospital for gradually worsening shortness of breath on minimal exertion, fatigue, persistent dry cough, lower extremity swelling of about two weeks duration. He did not have any history of hypertension or family history of heart disease. His HIV treatment regimen consisted of zidovudine, nevirapine and efavirenz. On admission he was alert and awake, but complained of mild respiratory distress. His vital parameters were: heart rate at 110 beats per minute and a blood pressure of 102/83 mmHg. There was elevated jugular venous pressure, displaced apex beat to the 6^th^ intercostal space, bilateral basal rales, and lower extremity edema. The heart sounds were faint, with frequent premature beats; there was no S3 and no murmurs. Relevant laboratory tests were within normal limits.

**Figure 1:**
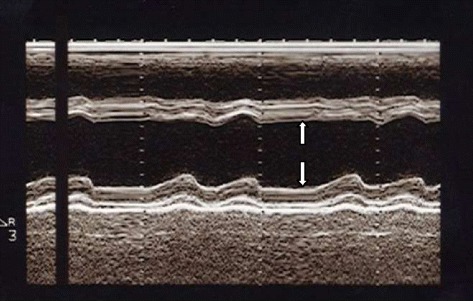
Parasternal long axis view at ventricular level showing left ventricular dilatation (left ventricular end diastolic diameter = 60 mm, white arrows).

**Figure 2:**
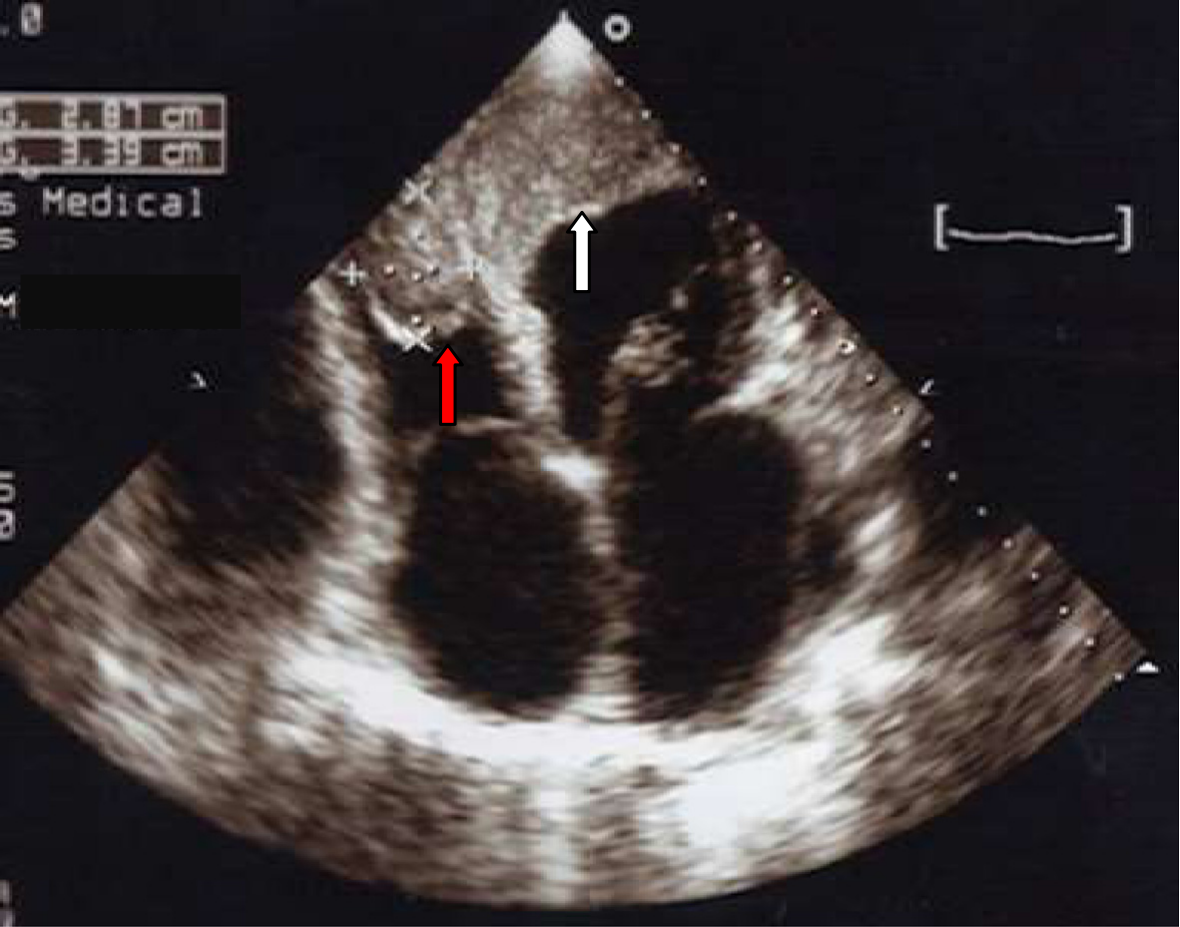
Apical four chamber view showing right ventricular thrombus (red arrow) and left ventricular thrombus (white arrow). The left ventricular thrombus is larger than the right.

**Figure 3:**
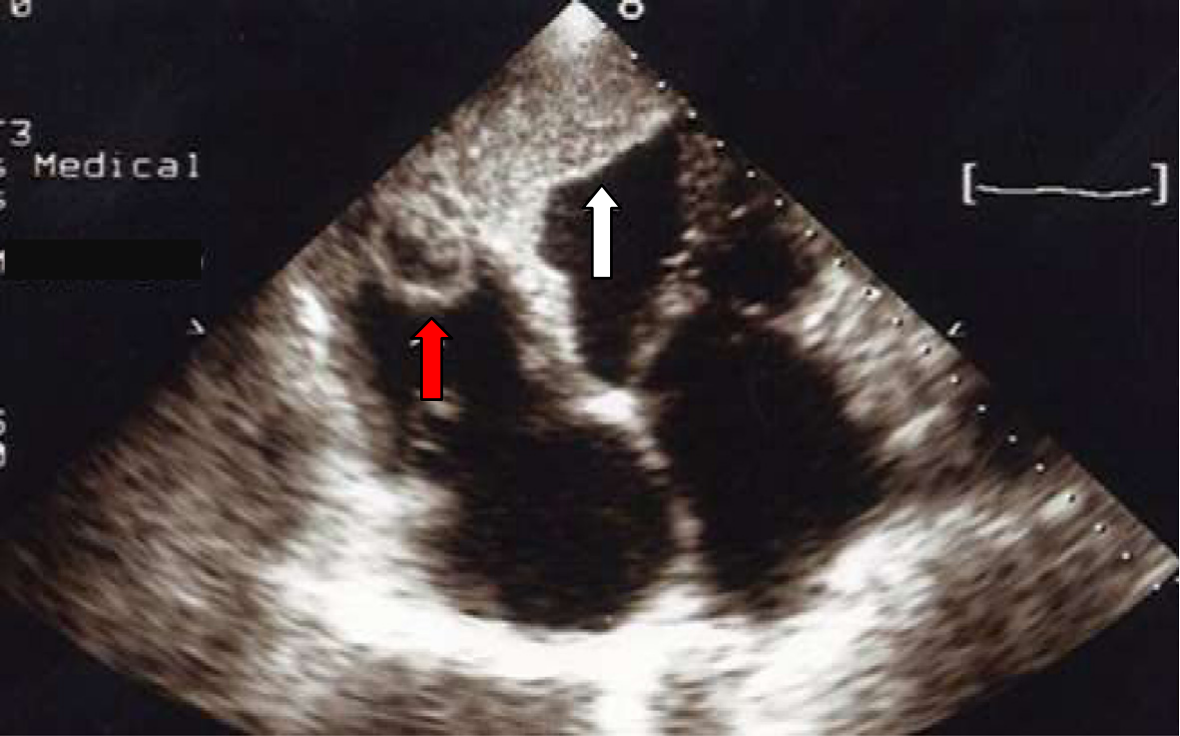
Apical four chamber view showing right ventricular thrombus (red arrow) and left ventricular thrombus (white arrow).

**Figure 4:**
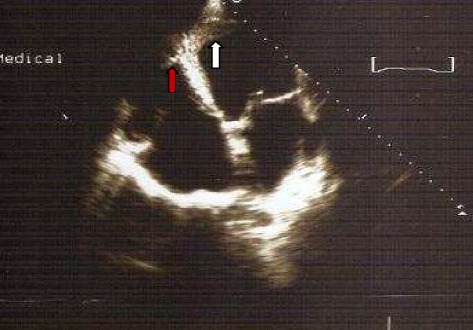
Apical four chamber view showing near complete dissolution of right ventricular thrombus (red arrow) and marked decrease in left ventricular thrombus (white arrow) after six months of anticoagulation.

Chest x-ray showed cardiomegaly, bilateral interstitial infiltrates. Serologies for hepatitis B and C were negative. He had a CD4 count of 135/mm^3^. The electrocardiogram showed sinus rhythm, poor R wave progression in precordials, with pathological Q waves in leads V1, V2, V3, V4, V5, leads I and aVL. Two-dimensional Echocardiography demonstrated a dilated left ventricle (Figure 1) with severe left ventricular systolic dysfunction (ejection fraction = 20%), with akinesis of the apex and, hypokinesis of the other segments of the left ventricle. There was mild mitral and severe tricuspid regurgitation, dilatation of the inferior vena cava as well as right and left ventricular masses (Figures 2 and 3). Both masses were dense than myocardium, acoustically distinct from underlying myocardium, well circumscribed, filling the apex of the left ventricle and the right ventricle. The right ventricular mass measured 28.7 mm × 33.9 mm while the left ventricular mass measured 29.5 mm × 45 mm. The masses were not mobile and prominent in the apical four chamber view. Bilateral ventricular thrombi were diagnosed. Cardiac catherization was not performed to explore the coronary anatomy. The diagnosis of dilated cardiomyopathy was made, complicated with biventricular thrombi in an HIV infected patient. There was no clinical evidence of thrombosis at other sites. Conventional treatment for congestive heart failure was started with loop diuretics and angiotensin converting enzyme inhibitors and he showed significant improvement of symptoms. Low molecular weight heparin was administered for the biventricular thrombi which was later on substituted with oral vitamin K antagonist (acenocoumarol) after overlapping with a target international normalized ratio (INR) between 2 and 3. The patient was discharged on day 12 on oral anticoagulation after he was reviewed by the HIV specialist. Six months later, a control echocardiography showed a near complete dissolution of the right ventricular thrombus and a marked decrease in the left ventricular thrombus (Figure 4); there was no evidence of neither pulmonary nor systemic embolization. Follow up INR were sub-therapeutic and the patient did not turn up for regular scheduled follow up visits to assess efficacy of anticoagulation and dose adjustments. There was no bleeding related to oral anticoagulation.

## Discussion

This paper reports on the observation of biventricular thrombi in dilated cardiomyopathy in an HIV infected patient with the risk of systemic and pulmonary embolization. The patient had other risk factors for developing dilated cardiomyopathy. He was a current cigarette smoker and heavy consumer of alcohol for more than ten years. With all these factors put together, dilated cardiomyopathy is likely to result. Heart muscle involvement associated with HIV infection can present as myocarditis, dilated cardiomyopathy or as isolated left or right ventricular dysfunction and coinfection with other infectious agents seems to play an important role in the pathogenesis [6]. A recent study in India showed that 17.6% of HIV infected patients have dilated cardiomyopathy [3]. It might be difficult to know the relative contributions of HIV infection, alcohol and cigarette smoking to the dilated cardiomyopathy in our patient as these are all known to cause dilated cardiomyopathy. Though no coronary angiography was performed in this resource limited setting where cardiac catherization laboratories are virtually non existent, the electrocardiogram and segmental wall motion abnormalities on echocardiography were suggestive of an associated ischemic cardiomyopathy. However, segmental wall motion abnormalities, even when left bundle branch has been excluded, are common in dilated cardiomyopathy in the absence of coronary artery disease [7].

To our knowledge, there are no previous reports of simultaneous left and right ventricular thrombi in dilated cardiomyopathy in patients with HIV infection from Cameroon and only few cases of biventricular thrombi have been reported elsewhere in SSA [5]. In Senegal, Damarou *et al.* reported biventricular thrombi complicating peripartum cardiomyopathy [5]. Friedman *et al.* for the first time reported the presence of biventricular thrombi on echocardiography following acute myocardial infarction [8]. Since then, other authors have reported biventricular thrombi in association with other conditions such as cocaine-Induced Myocardial infarction [9] and biventricular thrombi in association with peripartum cardiomyopathy [10,11]. The hypercoagulable state in HIV infected patients [12] associated with the severe biventricular systolic dysfunction in this patient with dilated cardiomyopathy most likely contributed to the development of the biventricular thrombi. Our patient did not have any evidence of systemic or pulmonary embolization as reported by some authors [13].

## Conclusion

Dilated cardiomyopathy may be seen in patients with HIV infection although other mechanisms needs to be assessed, but the occurrence of biventricular thrombi is rare.

### Consent

Written informed consent was obtained from the patient for publication of this Case Report and any accompanying images. A copy of the written consent is available for review by the Editor-in-Chief of this journal.

## Abbreviations

SSA: Sub-Saharan Africa
HIV: Human immunodeficiency virus infection
INR: International normalized ratio
